# Decreased and Increased Baroreceptor Sensitivity Are Associated With Incident Heart Failure in the Elderly: A 15‐Year Follow‐Up Study

**DOI:** 10.1002/clc.70316

**Published:** 2026-04-20

**Authors:** Bozena Ostrowska, Carina Blomström‐Lundqvist, Lars Lind

**Affiliations:** ^1^ Department of Medical Sciences Uppsala University Uppsala Sweden; ^2^ School of Medical Science, Faculty of Medicine and Health Örebro University Örebro Sweden

**Keywords:** alpha index, baroreceptor sensitivity, baroreceptors, incident heart failure, sequence analysis, spectral analysis

## Abstract

**Aims:**

Congestive heart failure (CHF) is associated with increased mortality. Early identification of individuals at risk for CHF may improve the poor prognosis. Decreased baroreceptor sensitivity (BRS) has been related to higher mortality in CHF. The aim was therefore to explore whether decreased BRS could identify patients at risk for the development of CHF.

**Methods and Results:**

The PIVUS (Prospective Investigation of the Vasculature in Uppsala Seniors) study (1016 individuals, all aged 70 years) was used for analysis of baroreceptor sensitivity, measured by sequence analysis (BRS_seq_) and spectral analysis (BRSαHF and BRSαLF). During 15 years of follow‐up, 98/844 individuals developed CHF. Both decreased and increased BRS_seq_ were associated with incident CHF (*p* = 0.027) after multiple adjustments. A similar pattern was seen for BRSαHF (*p* = 0.017) in a sex‐adjusted model, but not after multiple adjustment, while BRSαLF was unrelated to incident CHF.

**Conclusion:**

A decreased BRS was associated with incident CHF in an elderly population. An increased BRS was also found, although to a lesser degree, to be linked to CHF, which was a novel finding. If reproduced in further studies, BRS might prove to be useful for an early identification of CHF in clinical practice.

## Graphical abstract

The PIVUS study was used for analysis of baroreceptor sensitivity, measured by sequence analysis (BRS_seq_) and spectral analysis (BRSαHF and BRSαLF). During 15 years of follow‐up, 98/844 individuals developed CHF. Both decreased and, to a lesser degree, increased BRS_seq_ and BRSαHF were associated with incident CHF, while BRSαLF was not.

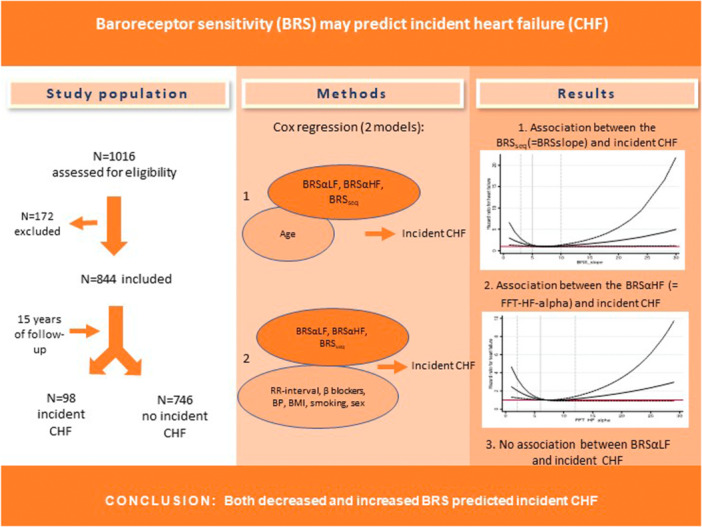

## 1 Introduction

Congestive heart failure (CHF) is an epidemic disease state with increasing prevalence mainly due to aging of the population [1], but also to an unhealthy lifestyle. Improved treatment strategies of CHF have resulted in improved survival [2].

The latest ESC and AHA/ACC/HFSA guidelines emphasize the importance of identifying and treating risk factors for developing CHF in order to prevent its onset [3, 4]. A challenge is, however, to identify asymptomatic individuals with an inherent risk of developing CHF but without clinically overt risk factors for CHF.

Heart failure is defined as a structural or functional cardiac abnormality accompanied by typical symptoms, signs of a failing myocardial function, or a change in biomarkers [3]. It is also a state of autonomic nervous system (ANS) imbalance with a sympathetic overactivity and a parasympathetic withdrawal regardless of the left ventricular ejection fraction [5]. The sympathetic predominance occurs early in the course of CHF [6] and is enhanced by concomitant comorbidities such as obesity, diabetes, renal impairment, or hypertension [7]. The persistent activation of the sympathetic nervous system (SNS) along with downregulation of the β‐receptors contributes to progressive impairment of the left ventricular systolic function and cardiac remodeling [8]. The observed sympathetic overactivity in CHF has been correlated with an increased mortality and a lower functional capacity [9]. Data on the ANS balance before development of CHF are scarce, but a decreased low frequency/high frequency (LF/HF) ratio [10], an increased variability of the R‐wave amplitude [11], along with a short P‐wave duration [12], each one of these variables indicating an ANS dysregulation, has been associated with incident CHF.

The activity of the ANS can be assessed by analyzing the heart rate variability (HRV) or the sensitivity of the arterial baroreceptor‐heart rate reflex. Several noninvasive methods are available for assessment of the baroreflex sensitivity (BRS) [13]. The “spontaneous BRS” techniques, based on spontaneous variations of systolic blood pressure and heart rate [14], have most frequently been used in clinical studies [15]. A decreased BRS has been associated with myocardial infarction (MI), chronic CHF, hypertension, diabetes, and coronary artery disease [16] as well as ventricular arrhythmias and higher mortality in chronic CHF [16, 17]. However, a relationship between BRS and incident CHF is unclear.

Given this background, we aimed to investigate whether changes in spontaneous BRS were related to the development of CHF in an elderly population.

## 2 Methods

### 2.1 Study Population

The present study was based on the PIVUS (Prospective Investigation of the Vasculature in Uppsala Seniors) study, which started in 2001. Individuals eligible for the PIVUS study were men and women living in the community of Uppsala, Sweden. In a randomized order, 2025 subjects were chosen from the community register and invited within 2 months following their 70th birthday [18]. Of the 2025 invited subjects, 1016 (50% women) consented.

The present study was performed on 844/1016 PIVUS participants after excluding those with a prevalent diagnosis of CHF, implanted pacemaker/defibrillator, atrial arrhythmias including atrial fibrillation (AF) diagnosis, second‐ and third‐degree atrioventricular block, delta waves, and QRS duration ≥ 130 ms.

Characteristics of the study population are presented in Table 1.

**Table 1:** Baseline characteristics in 844 individuals.

Variables	Values
Female sex, *n* (%)	418 (49.5)
Smoker, *n* (%)	84 (10)
BMI (kg/m^2^)	27 (4.2)
Beta‐blockers therapy, *n* (%)	170 (20)
Systolic blood pressure (mmHg)	150 (18)
Use of antihypertensive treatment, *n* (%)	250 (30)
Diabetes, *n* (%)	95 (11)
AF, *n* (%)	148 (21)
MI, *n* (%)	92 (11)
LVEF (%)	67 (6)
RR‐interval (ms)	980 (136)
BRS sequence^a^ (ms/mm Hg)	6.2 (3.4)
BRSαLF (ms/mm Hg)	7.1 (3.8)
BRSαHF (ms/mm Hg)	6.5 (4.3)

*Note:* Figures are means and 1 standard deviation (SD) unless otherwise stated.

Abbreviations: AF, atrial fibrillation (occurring between baseline and CHF diagnosis); BMI, body mass index; BRS, baroreflex sensitivity; BRSαHF, BRS α index in HF; BRSαLF, BRS α index in LF; Hg, mercury; HF, high frequency; IQR, interquartile range; LF, low frequency; LVEF, left ventricular ejection fraction; MI, myocardial infarction (occurring between baseline and CHF diagnosis).

^a^ 9 missing values.

### 2.2 Study Design

Prior to the baseline examination, the PIVUS participants completed a questionnaire concerning their medical history, medications, and smoking habits. A baseline cardiovascular (CV) examination was performed in the morning after an overnight fast. No coffee, smoking, or medications were allowed on the day of the examination, which included blood pressure, electrocardiography (ECG), echocardiographic examination, and blood sampling for biomarkers.

The systolic arterial blood pressure (SAP) was recorded invasively via an intra‐arterial catheter in the brachial artery. The six precordial ECG leads (V1 through V6) were recorded for 5 min in a quiet room at a constant temperature, in supine resting position and with controlled breathing (12/min = 0.2 Hz). The ECG and SAP signals were digitized at a sampling rate of 500 Hz. Each ECG recording was manually edited, and artifacts such as ectopic beats or triggering unmarked R‐peaks were removed. Subjects with technically inadequate recordings (*n* = 9) or ongoing AF were excluded, leaving 835 individuals for analysis of BRS with sequence method, while 844 individuals were analyzed with spectral method.

The time series of RR and SAP were generated. The RR‐intervals were measured as the time distance between two consecutive R‐wave peaks. The SAP was measured as the maximum of arterial pressure in the corresponding RR‐interval. Two separate methods were employed for the calculation of BRS.

### 2.3 Sequence Analysis

A series of ≥ 3 consecutive cardiac cycles with SAP and the following RR interval progressively increasing or decreasing in the same direction by ≥ 1 mmHg/beat and ≥ 3 ms/beat, respectively [13, 19], were defined as sequences. The sequences were identified by a custom‐made software (based on Excel; Microsoft Inc.), which scanned the ECG data. The slope of the regression line between the SAP (independent variables) and the RR intervals (dependent variables) for each sequence was estimated. A mean value of the slopes was calculated and defined as BRS_Seq_.

### 2.4 Spectral Analysis

Spectral analysis of SAP and RR‐interval variations was performed by a proprietary software (Ekman Biomedical Data AB; Gothenburg, Sweden [using MATLAB; Math‐Works Inc; Natick, MA]). The signal was linearly detrended, and a low‐pass filter of 1 Hz was applied. The power spectrum density of the signal was calculated using the fast Fourier transform (the Welch algorithm). The data were divided into two blocks with a 50% overlap, and each block was windowed using the Hamming function. The BRS α indices (BRSαLF and BRSαHF) were calculated separately in the LF band (0.03–0.15 Hz) and the HF band (0.15−0.25 Hz), respectively, as the square root of the ratio of the power of RR variability to the power of SAP variability in the same band.

In the PIVUS population, all examinations were repeated at the ages of 75 and 80 years, except for the ECG analysis, which was limited to assessing AF only. The first clinical diagnosis of CHF, made in‐hospital on the basis of symptoms, clinical status, BNP, chest X‐ray, and in some cases ECHO, and consistent with the ESC CHF guidelines, was defined as incident CHF. Data on CHF, AF, and MI diagnoses of the PIVUS study participants were retrieved from the Swedish Cause of Death Register and the Swedish Hospital Discharge Register, both with high quality and accuracy [12]. All retrieved diagnoses were validated by an experienced internist regarding their alignment with the guidelines. A detailed description of the PIVUS study has been published elsewhere [12, 18, 20].

The Ethics Committee of the University of Uppsala approved the entire PIVUS study. Written informed consent was obtained from each participant before the start of the study [21].

### 2.5 Statistical Methods

Cox proportional hazard analysis was used to relate the BRS indices to incident CHF. The BRS indices were modeled as a restricted cubic spline function with three knots (10th, 50th, and 90th percentiles) due to an assumption of nonlinear relationships.

In the first set of models, adjustment was performed for sex (age same in all subjects). In the second set of models, adjustment was performed for RR‐interval, medication with β‐blockers, systolic blood pressure, body mass index (BMI), and smoking. Lipids and diabetes were not related to incident CHF in initial models regarding confounders, and were therefore not used as confounders in the present analysis.

In order to test if there was a sex‐difference regarding the relationship between BRS and incident CHF, we introduced an interaction term between sex and BRS (spline function).

A *p* < 0.05 was considered significant. STATA16 (Stata Inc., College Station, TX) was used for the calculations.

## 3 Results

During a median of 15 years of follow‐up, 98/844 (11.6%) individuals developed CHF.

**Figure 1:**
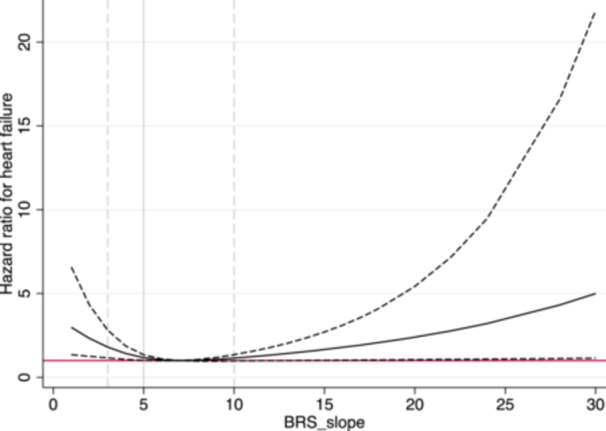
Relationship between baroreceptor sensitivity (sequence method) and incident heart failure. The solid line represents the hazard ratio for incident heart failure. The dashed line represents the 95% confidence interval. BRS‐slope, baroreceptor sensitivity calculated by the sequence method.

In the sex adjusted model (Figure 1), BRS calculated by the sequence method was significantly related to incident CHF (*p* = 0.013). This relationship was still significant after adjusting for SBP, BMI, smoking, β‐blocker treatment, and RR‐interval (*p* = 0.027). The relationship was nonlinear with a narrower confidence interval (CI) at low levels of BRS_Seq_ compared to the levels higher than the reference value = 7. A significant risk increase was seen already for the BRS_Seq_ = 5 (hazard ratio [HR] = 1.17, 95% CI 1.01−1.35). For BRS_Seq_ = 3, the HR was 1.81 (95% CI 1.16−2.82).

**Figure 2:**
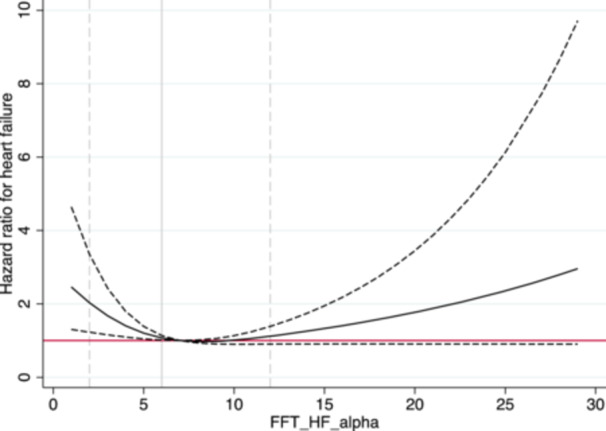
Relationship between baroreceptor sensitivity calculated by the spectral method in high frequency and incident heart failure. The solid line represents the hazard ratio for incident heart failure. The dashed line represents the 95% confidence interval. FFT‐HF‐alpha, baroreceptor sensitivity (α‐index) calculated by the spectral method in high frequency (HF) using fast Fourier transform (FFT).

**Figure 3:**
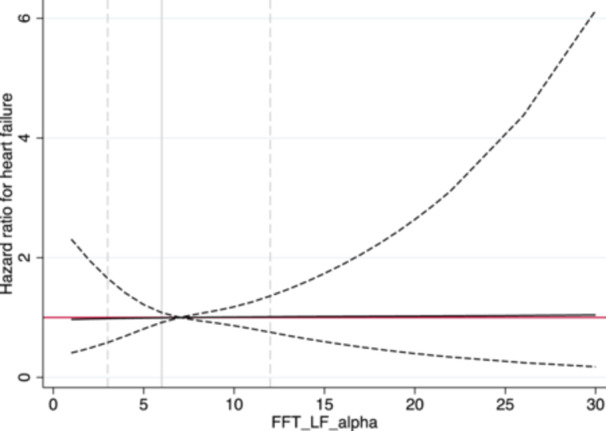
Relationship between baroreceptor sensitivity calculated by the spectral method in low frequency and incident heart failure. The solid line represents the hazard ratio for incident heart failure. The dashed line represents the 95% confidence interval. FFT‐LF‐alpha, baroreceptor sensitivity (α‐index) calculated by the spectral method in low frequency (LF) using fast Fourier transform (FFT).

A similar pattern of relationship with incident CHF was observed for BRS calculated by the spectral method in HF (BRSαHF) (Figure 2). In the sex‐adjusted model, BRSαHF was significantly related to incident CHF (*p *= 0.017), but this relationship was lost after adjustment for multiple confounders as above (*p *= 0.16). In the sex‐adjusted model, a slightly increased risk was seen already at BRSαHF = 6 (HR = 1.07, 95% CI 1.01−1.14). For the BRSαHF = 2 (10th percentile), the HR was 2.03 (95% CI 1.23−3.35). BRS calculated by the spectral method in LF (BRSαLF) was unrelated to incident CHF (Figure 3). The *p* value for the sex and BRS interaction term was 0.10, indicating that there was no significant sex difference.

**Figure 4:**
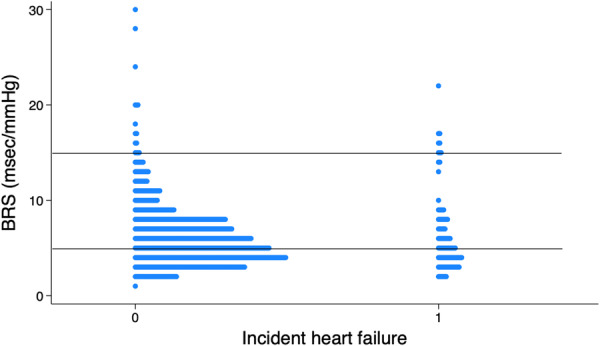
Distribution of the baroreceptor sensitivity values in the study population. Incident heart failure = 0 represents individuals without incident heart failure, and =1 represents individuals with incident heart failure. BRS, baroreceptor sensitivity.

Baseline low values of BRS (< 5 ms/mmHg) were more common than high values (> 15 ms/mmHg) in subjects with incident heart failure (Figure 4).

## 4 Discussion

A U‐shaped relationship was seen between BRS and incident CHF. This relationship was strongest at low BRS values, but was also observed for high BRS values.

Arterial baroreceptors are stretch receptors providing the central nervous system (CNS) with continuous information on the arterial blood pressure (ABP). An increase of the ABP activates the vagal cardioinhibitory mechanism and inhibits the SNS activity, resulting in bradycardia, decreased cardiac contractility, and lower peripheral vascular resistance. On the other hand, a decrease in ABP leads to vagal inhibition and activation of the SNS, resulting in tachycardia [16]. Therefore, the arterial baroreflex is one of the key mechanisms of homeostasis by stabilizing ABP and maintaining an adequate perfusion. CV diseases are often accompanied by a chronic adrenergic activation, which subsequently impairs the function of the baroreceptors [16]. Indeed, a reduced BRS has been observed in prevalent CHF, hypertension, and ischemic heart disease [16]. A BRS value of ≤ 3 ms/mmHg has been associated with arrhythmias and higher mortality in chronic CHF [16, 17]. Although a reduced BRS has also been associated with aging, the prognostic ability of the BRS has not been age dependent [22].

The present study found an association between a reduced BRS and incident CHF, which indicates a sympathetic dominance prior to the development of CHF. It was, however, difficult to determine whether this finding was due to a decreased vagal activity or an excessive sympathetic activation, and further exploration is beyond the scope of this study.

The association between increased BRS, suggesting enhanced vagal tone, and incident CHF was less pronounced (just above the limit of significance), yet highly unexpected, since increased vagal tone has been considered cardioprotective [23]. One explanation for this finding may be genetic factors. Race differences in the effectiveness of the baroreflex function have been described and termed the CV conundrum. When compared to European Americans, African Americans have higher total vascular resistance, higher blood pressure, and an increased rate of CV complications despite having enhanced vagal tone [23]. In another population‐based study, a genetic variation in the aldosterone‐synthase gene has been found to influence the BRS [24]. A genetically mediated enhanced BRS has also been associated with a high propensity for arrhythmias in patients with an IKs current mutation [25]. In another study, the heritability of BRS has been confirmed in twins [26]. Although the population of the present study was ethnically homogenic, genetic variations in the BRS were not unlikely. Another hypothetical explanation for the association between an increased BRS and incident CHF may be a vascular dysfunction, a feature of CV autonomic dysfunction [27]. One animal study has found a high BRS, possibly as a compensatory adaptation, in calponin knock‐out mice exhibiting a blunted alpha‐adrenergic vascular response to SNS activity [28].

The results of the present study were consistent for the BRS_seq_ and BRSαHF. However, there is no true gold standard for noninvasive measurement of the sensitivity of spontaneous baroreflex control of the heart rate [29]. The beat‐to‐beat‐based BRS reflects largely the vagal cardiac activity characterized by an immediate action on the heart rate [16] as opposed to gradual heart rate changes due to activation of the ANS. The sequence method provides information predominantly about the vagal cardiac activity, since the majority of the sequences consist of less than six RR intervals [13]. Calculation of the spectral BRSα indices, on the other hand, requires a time window of 128−256 beats [13]. The sequence analysis comprises a wide frequency range when compared to the relatively narrow frequency bands analyzed by the spectral methods. This may affect the results since the frequency range of the baroreflex‐driven sinus node modulation is wider than that analyzed by the spectral technique [13]. Although some authors have raised concerns regarding the accuracy of the sequence method [30], it has commonly been employed in clinical trials.

It is generally accepted that the HF power reflects modulation of heart rate predominantly by the vagal cardiac activity, but respiration may be a significant confounding factor [14]. In contrast, the origin of the LF component is unclear [31]. Despite the above differences, one study has found a strong relationship between the sequence analysis and the spectral analysis in the HF and LF, in a supine position of the patient [32].

There was no association between the BRSαLF and incident CHF in the present study. However, the value of the LF power of the HRV for measurement of the BRS is controversial. While some authors have found a strong correlation between the BRSαLF and clinical outcomes in CHF patients [14], others deemed the LF as unsuitable for measurement of the baroreflex function [33].

The strength of this study is the long follow‐up, which enabled the detection of the majority of new CHF cases. Another strength of the study is its design. The ABP was registered invasively, while other BRS studies have almost invariably implemented noninvasive ABP measurements, which are less reliable than the invasive method [15]. The ECG was recorded during controlled breathing, which has been found effective in removing abnormal breathing patterns without altering the autonomic regulation [34]. Furthermore, all ectopic beats were excluded since they could affect the BRS measurements [35].

A limitation was that a distinction could not be made between CHF with reduced and preserved ejection fraction, as the diagnosis of the latter was unclear at the time of data collection.

Since the increased risk seen at higher values was just above the limit of significance, it should be taken with caution until reproduced by others.

## Conflicts of Interest

Carina Blomstrom‐Lundqvist reports personal fees from Bayer, Medtronic, CathPrint, Philips, Sanofi Aventis, Boston Sci, Abbott, and Organon outside the submitted work. The other authors declare no conflicts of interest.

## Data Availability

The data underlying this article cannot be shared publicly due to Swedish law and the Ethical Committee's permission. The data may be shared based on a request to the corresponding author.
